# Acquired von Willebrand Syndrome in a 17-Year-Old With Essential Thrombocythemia: A Case Report With Literature Review

**DOI:** 10.7759/cureus.55668

**Published:** 2024-03-06

**Authors:** Linda Youn, Amber Kuta, Mirra Srinivasan, Renuka Mahatara, Mazen Khalil

**Affiliations:** 1 Internal Medicine, St. Bernards Medical Center, Jonesboro, USA; 2 Internal Medicine, New York Institute of Technology College of Osteopathic Medicine, Jonesboro, USA; 3 Hematology/Oncology, St. Bernards Medical Center, Jonesboro, USA

**Keywords:** jak 2 gene mutation, elevated platelet, bleeding, essential thrombocythemia, acquired von willebrand syndrome

## Abstract

Acquired von Willebrand syndrome (AVWS) is a rare bleeding disorder that is often underdiagnosed. AVWS typically occurs in adults without a family history of bleeding disorders and with associated conditions such as lymphoproliferative, myeloproliferative, and cardiovascular disorders. Here, we present a case of AVWS in a young patient with essential thrombocythemia and a literature review on AVWS in the setting of essential thrombocythemia.

## Introduction

Acquired von Willebrand syndrome (AVWS) is a rare disorder where patients are often asymptomatic, especially for decades, making it an underdiagnosed condition [1]. However, AVWS should be suspected in patients with an underlying condition that is associated with low von Willebrand factor (VWF) levels and new-onset unexplained mucocutaneous bleeding. There are a number of associated conditions with lymphoproliferative and myeloproliferative disorders making up the majority of those cases [2], and one of the most commonly associated disorders with AVWS is essential thrombocythemia (ET), a rare genetic disorder that causes thrombocytosis and bone marrow megakaryocytic hyperplasia due to several mutations [3] and carries a complicated course of both thrombotic and hemorrhagic events [4]. A retrospective study of 170 consecutive patients with ET, 20% were found to have concomitant AVWS [5].

AVWS in patients with ET raises serious considerations as prevention of thrombosis in individuals with ET could exacerbate the underlying bleeding risk associated with AVWS [1,6]. Thus, identifying ET patients who have an increased risk of also having AVWS will guide treatment and improve outcomes. It is also proved that aspirin will prevent episodes of thrombosis; however, patients with extremely high platelet count, defined as >1000×10⁹/L, and a ristocetin cofactor activity (RCo) <30% are at increased risk of bleeding if prescribed aspirin [4,6]. The purpose of this article is to emphasize the clinical implications of diagnosing AVWS in patients with underlying myeloproliferative disorders, especially ET and polycythemia vera (PV), in hopes of being aware of complicated bleeding episodes. Thus, screening for AVWS should be part of the routine assessment in ET and PV patients.

## Case presentation

Our patient is a 17-year-old male without significant past medical history who presented with complaints of dizziness, nausea, and vision changes. The initial workup was unremarkable, except for an elevated platelet count of 984 x 10⁹/L. MRI head was negative. The patient underwent a thrombocythemia workup. Abdominal ultrasound revealed a spleen size of 10.4 x 11.6 x 5.6 with slightly increased volume, indicating mild splenomegaly. A bone marrow biopsy showed a mildly hypercellular marrow (50%) containing trilineage hematopoiesis with complete maturation and increased megakaryocytes with megakaryocytic atypia in a pattern most consistent with essential thrombocythemia. No increase in blasts was noted. Genetic testing showed findings consistent with CALR+ myeloid neoplasm, negative JAK2, and negative BCR-ABL. Initially, the VWF was 44%, the ristocetin cofactor was 15%, and factor VIII activity was 33%, indicating AVWS secondary to ET (Table 1).

**Table 1:** Results of von willebrand factor (vWF), vWF risocetin cofactor, and factor VIII activity.

	von Willebrand factor activity	vWF Ristocetin cofactor activity	Factor VIII activity
Reference range	52-214%	51-215%	56-191%
1/19/23	44	15	33
3/3/23	47	33	34
6/9/23	79	29	42
1/5/24	50	55	55

The patient continued to experience vasomotor symptoms, including headaches, erythromelalgia, and transient visual disturbances. The patient was started on hydroxyurea, which provided symptom relief. Follow-up coagulation panels also showed improvement with vWF of 47%, ristocetin of 33%, and factor VIII activity of 34% (Table 1). Due to the continuation of fatigue, nausea, headache, and erythromelalgia, the dose of hydroxyurea was increased from 1,000 mg daily to 1,500 mg daily. The patient’s symptoms improved with the increased dose of hydroxyurea. The patient was also referred to a tertiary center for further evaluation and consultation. The tertiary center recommended stopping the hydroxyurea and continuing to monitor it. A repeat coagulation panel showed continued improvement in vWF, ristocetin cofactor, and factor VIII activities (Table 1).

## Discussion

AVWS is a heterogeneous bleeding disorder that has been historically underdiagnosed and should be a differential diagnosis in patients presenting with mucocutaneous bleeding in the setting of lymphoproliferative, myeloproliferative, and autoimmune disorders. As bleeding risk can be amplified by concomitant von Willebrand syndrome and ET, it is imperative to identify ET patients who have an increased risk of also developing AVWS to mitigate thrombosis risk. In this article, we present a case of ET with AVWS in a 17-year-old male, as well as a literature review of AVWS in the setting of ET.

Methodology

**Figure 1:**
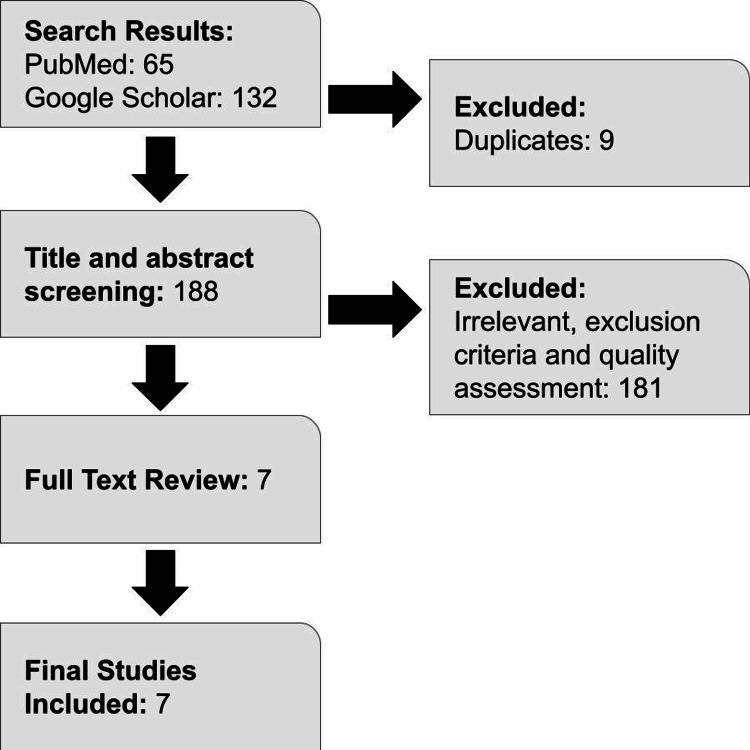
The flow chart provides a description of the studies selected for this literature review.

The quality of the studies selected was based on the scale for the assessment of narrative review articles (SANRA) checklist and included studies that scored 70% or more as final studies. The exclusion criteria included all studies in the non-English language, inaccurate data, and comorbidities that could bias the result (Figure 1).

Results

Table 2 summarizes the details of various studies included in this literature review and other observational studies (Table 3).

**Table 2:** Summarizes the results and conclusions of the included case report studies.

Authors/year	Case summary	Platelet count	Mutational analysis	Treatment
Pikta et al. [7]	A 33-year-old female with heavy menorrhagia and elevated platelet count	1391 x 10⁹/L	JAK2 (V617F) gene positive, CALR gene negative	Tranexamic acid during menstrual bleeding
	A 61-year-old female with bleeding post tooth extraction lasting for 2 days with high platelet count	1120 x 10⁹/L	JAK2 (V617F) gene positive, CALR gene negative	Hydroxyurea
Oyedeji et al. [8]	A 51-year-old female with recent multivisceral transplantation presents with bleeding from the oral cavity, GI tract, vagina	1512 × 10⁹/L	JAK2 (V617F) gene positive	Thrombocytapheresis (As an adjunct to bridging therapy before a response to hydroxyurea is achieved)
Kanderi et al. [9]	A 25-year-old male presented with acute chest pain. Diagnosed with MI, and LAD thrombotic occlusion with EF 25% on LHC, underwent thrombectomy and balloon angioplasty with LAD stenting. Developed significant post-op bleeding after receiving aspirin and ticagrelor.	1472 × 10⁹/L	Negative for JAK2, MPL, and CALR gene	Emergency plateletpheresis
Sasi et al. [10]	A 42-year-old male presented with gum bleeding, hemoptysis, no antecedent trauma reported	2300 x 10⁹/L	JAK2 (V617F) gene positive, CALR gene negative	Hydroxyurea
Schneider et al. [3]	A 14-year-old female patient fulminantly presented with acute symptoms comprising visual impairment, palmar and plantar stabbing pain.	2373 × 10⁹/L	JAK2V617F, CALR and MPL were negative	Hydroxycarbamide d/c due to neutropenia, switched to pegylated interferon
Rupa-Matysek et al. [11]	A 38-year-old male suffering from ET presented with two episodes of post-arthroscopic joint bleeding after synovectomy	638 × 10⁹/L	JAK2 (V617F) gene positive, MPL negative	Hydroxyurea
van Genderen et al. [12]	A 42-year-old with ET complicated by two major mucocutaneous bleeding episodes in a four-year period	>2000 x 10⁹/L on both occasions of the bleeding episodes		Hydroxyurea

**Table 3:** Summarizes other observational studies included in this review.

Author/year	Study objective	Results	Conclusions
Rottenstreich et al. [13]	To characterize AVWS among ET and polycythemia	Of 116 patients with ET, 64 (55%) developed AVWS; of 57 with PV, 28 (49%) developed AVWS. Of patients who developed AVWS, 69.5% had platelet counts below 1000×10^9^/L.	AVWS was common in ET and PV patients and associated with higher bleeding rates and higher platelet count and AVWS screening should be included in routine assessment for these patients.
Mital et al. [5]	To determine the prevalence of AVWS in patients with ET	Of 170 patients with ET, 34 (20%) were found to have AVWS. In addition, these patients had higher red blood cell and platelet counts and showed abnormal coagulation profiles.	All patients with ET and signs of a bleeding disorder, irrespective of the platelet count, should be tested for the presence of AVWS.

Essential thrombocythemia is a philadelphia-negative myeloproliferative neoplasm characterized by an increased platelet count. The World Health Organization (WHO) updated its diagnostic criteria in 2016, which requires all four major criteria or three major and one minor for the diagnosis to be established. The major criteria include a platelet count greater than or equal to 450 × 10^9^/L, a BM biopsy with increased megakaryocytes with hyperlobulated nuclei, no significant neutrophil myelopoiesis or erythropoiesis, and grate 1 increase in reticulin fibers, must not meet the WHO criteria for other myeloid neoplasms, and the presence of JAK2, CALR or MPL mutation. The minor criteria consist of clonal marker presence or absence of evidence of reactive thrombocytosis [1]. The JAK2 (V627F) mutation is most common with approximately 50-60% of cases found to have the mutation, followed by CALR with an estimated 25-35% mutation and MPL mutation with an approximately 5-10% mutation rate.

CALR-mutated ET is very different from JAK2 (V617F) ET. An analysis of 1,235 patients diagnosed with ET or PT by Rumi et al. [2] revealed that patients with CALR-mutated ET were significantly younger than those with JAK2-mutated ET [10]. In terms of mutant allele burdens, CALR-mutated ET rarely has a greater than 75% burden, while JAK2-mutated ET has a high burden [1]. Phenotypically, CALR-mutated ET seems to be associated with platelet counts in the thousands, but have a lower risk of thrombosis [1]. Platelets of CALR mutant patients were significantly less activated following adenosine diphosphate (ADP) stimulation compared to the control group and JAK2 mutants [14]. Seemingly, these observations all support the theory that granulocyte and platelet activation with increased white blood cell count may drive the pathogenesis of thrombotic complication rather than platelet count [2]. Rottenstreich et al. [3] found that JAK2 V617F was strongly associated with the development of acquired von Willebrand syndrome among ET patients.

AVWS is a rare bleeding disorder that can be characterized by qualitative, structural, or functional disorders of VWF that are secondary to other disorders, such as autoimmune, myeloproliferative, lymphoproliferative, etc. This leads to increased bleeding risk; thus, goals in care reflect controlling acute bleeding, preventing bleeding in high-risk situations, and remission. Therapeutic options can vary based on the underlying disorder; however, treatment of the underlying disorder does not guarantee improvement of AVWS [15].

As in the case presented, patients with ET and AVWS present a challenge in managing thrombotic and bleeding episodes. The international prognostic score of thrombosis in the WHO-essential thrombocythemia (IPSET-Thrombosis) is based on age, history of thrombosis, cardiovascular risk, and JAK2V617F mutation and stratified into low, intermediate, and high risk. This model provides an estimate of the probability of thrombotic events in patients with ET and is an important tool in guiding treatment as the primary objective of management is to prevent thromboembolic complications [16,17]. In cases of ET, a platelet count greater than one million is a risk factor for bleeding; however, these patients may also be at an increased risk for both arterial and venous thrombosis. The mainstay treatment of ET consists of low-dose aspirin and cytoreductive therapy. Ruxolitinib, pipobroman, and apheresis are alternative approaches to cytoreductive treatment resistance or intolerance. Treatment of AVWS is not always indicated; however, when bleeding difficulties arise or in preparation for surgery, hemostatic therapies such as desmopressin (DDAVP), vWF/FVIII, antifibrinolytics, etc. can be deployed [18].

## Conclusions

CALR-mutated ET is a myeloproliferative neoplasm that affects young individuals, characterized by markedly elevated platelet count and relatively low thrombotic risk. However, approximately 20% of patients with ET may also develop AVWS, which increases the risk of bleeding, complicating the risk of thrombosis that is also present. Therapeutic options are varied and aimed at preventing bleeding while balancing for risk of thrombosis and sustaining hematologic remission. Thus, we encourage routine screening for acquired von Willebrand syndrome in patients with ET and signs of bleeding.

## References

[1] Awada H, Voso MT, Guglielmelli P, Gurnari C (2020). Essential thrombocythemia and acquired Von Willebrand syndrome: the Shadowlands between thrombosis and bleeding. Cancers (Basel).

[2] Rumi E, Pietra D, Ferretti V (2014). JAK2 or CALR mutation status defines subtypes of essential thrombocythemia with substantially different clinical course and outcomes. Blood.

[3] Schneider C, Stutz-Grunder E, Lüer S (2019). Fulminant essential thrombocythemia associated with acquired Von Willebrand syndrome and bleeding episodes in a 14-year-old girl. Hamostaseologie.

[4] Michiels JJ (1999). Acquired von Willebrand disease due to increasing platelet count can readily explain the paradox of thrombosis and bleeding in thrombocythemia. Clin Appl Thromb Hemost.

[5] Mital A, Prejzner W, Bieniaszewska M (2015). Prevalence of acquired von Willebrand syndrome during essential thrombocythemia: a retrospective analysis of 170 consecutive patients. Pol Arch Med Wewn.

[6] Lancellotti S, Dragani A, Ranalli P (2015). Qualitative and quantitative modifications of von Willebrand factor in patients with essential thrombocythemia and controlled platelet count. J Thromb Haemost.

[7] Pikta M, Banys V, Szanto T (2021). Von Willebrand factor multimeric assay in acquired Von Willebrand disease diagnosis: a report of experience from North Estonia Medical Centre. J Lab Physicians.

[8] Oyedeji O, Sheqwara J, Onwubiko I, Lopez-Plaza I, Nagai S, Otrock ZK (2021). Thrombocytapheresis for acquired von Willebrand syndrome in a patient with essential thrombocythemia and recent multivisceral transplantation. Transfusion.

[9] Kanderi T, Puthenpura M, Shrimanker I, Sapna F, Felter SC (2020). Triple-negative essential thrombocythemia complicated by thrombosis and acquired Von Willebrand disease in a young man. Am J Case Rep.

[10] Sasi S, Yassin MA, Fadul AM (2020). A case of acquired von Willebrand disease secondary to myeloproliferative neoplasm. Case Rep Oncol.

[11] Rupa-Matysek J, Lewandowski K, Lewandowska M (2015). Bleeding complications after arthroscopy in a JAK2V617F-positive patient with essential thrombocythemia and acquired von Willebrand syndrome (AVWS). Int J Hematol.

[12] van Genderen PJ, Michiels JJ, van der Poel-van de Luytgaarde SC, van Vliet HH (1994). Acquired von Willebrand disease as a cause of recurrent mucocutaneous bleeding in primary thrombocythemia: relationship with platelet count. Ann Hematol.

[13] Rottenstreich A, Kleinstern G, Krichevsky S (2017). Factors related to the development of acquired von Willebrand syndrome in patients with essential thrombocythemia and polycythemia vera. Eur J Intern Med.

[14] Hauschner H, Bokstad Horev M, Misgav M, Nagar M, Seligsohn U, Rosenberg N, Koren-Michowitz M (2020). Platelets from Calreticulin mutated essential thrombocythemia patients are less reactive than JAK2 V617F mutated platelets. Am J Hematol.

[15] Tiede A, Rand JH, Budde U, Ganser A, Federici AB (2011). How I treat the acquired von Willebrand syndrome. Blood.

[16] Barbui T, Finazzi G, Carobbio A (2012). Development and validation of an International Prognostic Score of thrombosis in World Health Organization-essential thrombocythemia (IPSET-thrombosis). Blood.

[17] Mancuso S, Accurso V, Santoro M (2020). The essential thrombocythemia, thrombotic risk stratification, and cardiovascular risk factors. Adv Hematol.

[18] Franchini M, Mannucci PM (2020). Acquired von Willebrand syndrome: focused for hematologists. Haematologica.

